# Dowieism and Eddyism

**Published:** 1907-01

**Authors:** 


### "DOWIEISM" AND "EDDYISM."

---

Reforms, as a rule, go in waves. Crying need induces a spirit of unrest that develops so slowly that it is often unsuspected by those most involved in it; but when its hour is at hand, there is no power to check its force. Investigation is the keystone of the Twentieth Century reform—inquiry and the white light of public opinion are the modern means that work out the end. The success of reform measures has been more far reaching in direct and indirect lines than even the most fanatical have dreamed. Reform is contagious and it is not surprising, after the thorough airing given to the rottenness of Dowie and Zion, that an eager, restless public spirit should soon notice discrepancies and penetrate the internal workings of *Eddyism* and *Pleasant View*. If the New York *World* is to be credited, the conditions in the headquarter of *Christian Science* send forth as vigorous and noxious a stench as ever came from the wreckage of *Dowie's* Christian Catholic Church. Accoridng to the newspaper dispatches, *Mrs Mary Baker Glover Eddy,* (each surname, by the way, represents a husband), is now suffering from cancer and, incidentally, is under the care of a Boston cancer specialist. Further, it is held that *Mrs. Eddy* has, for three years past, been impersonated by another and a younger woman who makes all her semi-public appearances for her. Then—and this is by no means novel or surprising, in view of other personally conducted religious cults—we are told that she has done away with a fortune of from fifteen to twenty millions of dollars, collected by her devoted followers throughout the land. In seeking aid from a cancer specialist, it is not the first time that *Mrs. Eddy* has given the lie to her professions of faith in the practice of that religion which she has preached with such phenominal success. She has, for a long time, hired physicians, dentists and surgeons. She saw to it that her grand-children were vaccinated and paid the fees for a surgical operation on her sister-in-law.

To those who have studied the rise and fall of cults, the disclosures concerning *Eddyism* are but natural. People of all ages have ever been susceptible to fakirs who come clothed in the cloak of religion. Curiosity, unrest and moral instability are impulses that, in the hands of a strong character and a magnetic personality, hurry idle listeners into active fellowship. As a rule, these new teachers, no matter how sincere their professions may be, have preyed upon the illiterate and the unread. The natural reaching out of human beings for some form of faith and the interest in the present day for such a faith as may be flavored with the occult, is very strong. *Dowieism* and numerous other isms have satisfied those of meagre education, but could not attract those who boast of superior intellect. *Mrs. Eddy* has created a cult to meet the demand for a high class article, and as from its first invitation, Christian Science flatters its fol-ers as well as promises health to body and soul, the enterprises was an instantaneous success. The idea was kept contantly to the fore that Christian Science could flourish only upon fertile ground of able mentality that those who did not believe were lacking in this fertile soil. Together with this attractive idea, and essential to the business end of the enterprise, the Biblical phrase, "*The servant is worthy of his hire*"—was burnished in gold upon the escutcheon of the cult, and it was in real gold. Soon the promoters of Christian

Science reveled in luxury—luxury purchased by the dollars of the aristocrat. Dowey reveled in luxury paid for by the plebian. Mrs. *Eddy's* was the refined luxury of education—*Dowie's* the gaudy luxury of the illiterate. Then came the itching palm that has caused many such cults to fall. *Dowie* openly appropriated the money of his people to advance his gigantic enterprises. He worked like a pirate—crudely, openly. Quietly the millions collected by Christian Scientists disappeared from *Mrs. Eddy s* strong box. There was no show or bluster about it. *Dowie* seems to have grasped his unholy dollars for himself. *Mrs. Eddy* is said to have permitted her spoils to go as legitimate dividends to those clever business associates who had capitalized her, promoted her and brought her from a career of turbulent and unsuccessful matrimonial exploits to her exalted place.

There is now an opening for a good, energetic organizer of a new sect; one who will be satisfied with fair momentary returns for his labors. We are firmly convinced that a splendid organization built on the lines of Christian Science, can be established and, regardless of its fakery, can be maintained indefinitely, provided the hand of the leader will retain an honest grip on the helm without regard for the palm which itches for illicitspoils.—*Ed. Chicago Clinic and Pure Water Journal*, November, 1906.

